# Comparative Analysis of Quantum-Mechanical and Standard Single-Structure Protein–Ligand Scoring Functions with MD-Based Free Energy Calculations

**DOI:** 10.1021/acs.jcim.5c00604

**Published:** 2025-07-19

**Authors:** Mehran Jalaie, Jindřich Fanfrlík, Adam Pecina, Martin Lepšík, Jan Řezáč

**Affiliations:** † 351664 Pfizer Worldwide Research and Development, Oncology Medicinal Chemistry, Pfizer, La Jolla, California 92121, United States; ‡ 89220 Institute of Organic Chemistry and Biochemistry of The Czech Academy of Sciences, 160 00 Prague, Czech Republic

## Abstract

Single-structure scoring functions have been considered inferior to expensive ensemble free energy methods in predicting protein–ligand affinities. We are revisiting this dogma with the recently developed semiempirical quantum-mechanical (SQM)-based scoring function, SQM2.20, comparing its performance to the standard scoring functions on one hand and state-of-the-art molecular dynamics (MD)-based free-energy methods on the other hand. The comparison is conducted on a well-established Wang data set comprising eight protein targets with 200 ligands. The initial low correlation of SQM2.20 scores with the experimental binding affinities of *R* ^2^ = 0.21 was improved to *R* ^2^ = 0.47 by a systematic refinement of the input structures and omission of the ligand deformation energy. Consequently, SQM2.20 representing accurate single-structure scoring functions, exhibited an average performance comparable to that of MD-based methods (*R* ^2^ = 0.52) and surpassed the performance of standard scoring functions (*R* ^2^ = 0.26). The per-target analysis highlighted the pivotal role of high-quality input structures on the outcomes of single-structure methods. In the instances where such structures are available, SQM2.20 scoring has been shown to rival or even exceed MD-based methods in predicting protein–ligand binding affinities, while exhibiting significantly reduced computation time.

## Graphical abstract

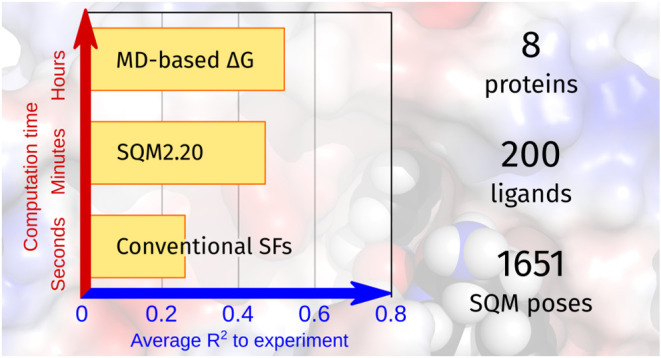

## 1 Introduction

The pursuit of structure-based computational protocols for computer-aided drug design represents an enticing yet formidable research endeavor.^1,2^ Accurate prediction of protein–ligand (P–L) affinities is essential, as the binding free energy window typically spans just a few kcal/mol. Target proteins comprise thousands of atoms, and the number of ligand variants to be assessed can easily reach the hundreds. It is a challenge to meet these two criteria, high accuracy and computational efficiency, at the same time.

Structure-based methods for estimating P–L binding affinity can be broadly divided into single-structure methods, which rely on a single structure of the P–L complex, and molecular dynamics (MD) simulations, which provide sampling of the system’s configuration space. Single-structure approaches range from fast scoring functions (SFs) used in docking to highly accurate but computationally demanding quantum mechanics (QM) calculations. These approaches often omit some contributions to the binding free energy, Δ*G*_bind_, referring to the computed quantity as a “score”. In contrast, MD-based protocols allow for a more rigorous calculation of the binding free energy from a statistical thermodynamics perspective, although they may be limited by the accuracy of the underlying molecular mechanics (MM) force field and the stochastic error due to imperfect sampling.

In this paper, we compare these two approaches to computing P-L affinities. In order to include a broad range of methods, we use the data set introduced by Wang et al. in 2015,^3^ for which the results of multiple MD-based free energy methods are now available in the literature.^4–9^ Accurate single-structure scoring is represented by the SQM2.20 scoring function based on semiempirical quantum-mechanical (SQM) calculations.^10^ We also include multiple standard SFs, SFs augmented with machine learning (ML) components, stand-alone structure-based ML approaches, and several other related methods.

Recent advancements in QM methods and computer hardware have facilitated the application of QM-based methods in the computer-aided drug design field.^11,12^ SQM2.20 represents a novel SQM-based SF^10^ which relies on PM6-D3H4X,^13–15^ combined with the implicit COSMO2 ^16^ solvation. It incorporates the latest methodological developments in the description of noncovalent interactions^17,18^ at the SQM level. The accuracy of PM6-D3H4X has been meticulously evaluated and compared with other methods in smaller models of P-L complexes, where reliable benchmark calculations are available.^19^ Moreover, its performance has been juxtaposed with density functional theory (DFT) in larger systems^10^ and in the structural description of proteins.^20^ Using the linear-scaling MOZYME algorithm^21^ instead of the standard self-consistent field procedure, these SQM calculations remain computationally efficient even for systems with thousands of atoms. SQM2.20 had been successfully validated using the PL-REX benchmark data set,^10^ which consists of high-resolution crystal structures (1.0–2.3 Å range for the structures selected for calculations, see Table S1 in the Supporting Information) and reliable experimental affinities for ten diverse protein targets and their associated ligands. In this data set, SQM2.20 outperformed commonly used SFs, achieved a level of accuracy similar to much more expensive DFT calculations, with excellent correlation with the experimental binding data (average squared Pearson’s coefficient, *R* ^2^, of 0.69), and had consistent performance across 10 targets.^10^

The Wang data set encompasses binding data for 8 protein targets and 200 ligands. Unlike PL-REX, it is supported by a mere 22 crystal structures with medium resolution (1.5–3.5 Å range). This implies that the success of binding affinity prediction within this set hinges not only on the quality of the computational methods employed, but also on the precision of the modeled structures of the P–L complexes used as input.

One of the key advantages of MD-based free energy calculations is their ability to capture the dynamics of the system and encompass all components of the binding free energy, Δ*G*_bind_. However, the extensive sampling of the configuration space, which often requires multiple windows and independent simulations per window to achieve converged results, renders these simulations computationally intensive. This level of demand is only feasible with empirical MM force fields, whose accuracy can be limited. On the other hand, more accurate QM calculations, including SQM2.20, typically compute only the leading contributions to Δ*G*_bind_ within a static model of the P–L complex. Given that the requirements for extensive sampling and accuracy of the potential are mutually exclusive, this study also offers a broader evaluation of the inherent strengths and limitations of these different approaches.

## 2 Methods

### 2.1 Wang Data Set

The general characteristics of the Wang data set are summarized in Table 1. In the original publication,^3^ only one PDB code per target was provided, without the full set of structures of the complexes. Later, Zariquiey et al. published a complete set of modeled P–L complexes^9^ (we will refer to them as “Zariquiey structures”). More recently, Ross et al. released another set of models when the original data set became a part of larger database (where it is designated as the “R-group”).^4^ We will refer to them as “Ross structures”; they differ in ligand placement and for JNK1 and MCL1 targets also include alternative poses of ligands (see Table S2 in the Supporting Information).

**Table 1:** Composition and Features of the Wang Dataset of Protein-Ligand Complexes^a^

target	protein	ligands/crystals	pKi range	ligand similarity	crystal used/resolution/RSCC
BACE	β-secretase 1	36/1	3.5	0.71	4DJW/1.9/0.91
CDK2	cyclin-dependent kinase 2	16/6	4.2	0.84	1H1Q/2.5/0.93
JNK1	c-Jun N-terminal kinase	21/1	3.4	0.85	2GMX/3.5/n.d.
MCL1	myeloid cell leukemia 1	42/1	4.2	0.67	4HW3/2.4/0.90
p38	p38 kinase	34/7	3.8	0.77	3FLY/1.8/0.86
PTP1B	phosphatase PTP1B	23/4	5.1	0.79	2QBS/2.1/0.98
thrombin	thrombin	11/1	1.7	0.84	2ZFF/1.5/0.96
Tyk2	TYK2 Kinase	16/1	4.3	0.84	4GIH/2.0/0.92

a The ligand similarity is expressed as the average of the Tanimoto coefficients computed for each pair of ligands. Crystallographic resolution is in Å. The real-space correlation coefficient (RSCC) of the ligands ranges from 0 to 1 and indicates the reliability of the model with respect to the crystallographic electron density.

The Zariquiey and Ross structure sets were used as input for SQM2.20 scoring. The Zariquiey structures needed some preparation and corrections: Unphysical hydrogen positions were corrected in ligands 23,477, 23,479, and 23,482 of PTP1B. Hydrogen atoms were added to the proteins using the Leap tool of the AMBER20 suite.^22^ In contrast, the Ross protein structures already contained correctly positioned hydrogens and were used without modification. There had been a controversy in the literature on the protonation state of Cys215 in PTP1B. Based on the recommendation of Gapsys et al.,^8^ we considered it protonated, as this state is compatible with the nearby carboxylate group of the ligands.

### 2.2 Expanding the Ligand Conformation Set

The input structures that yielded a superior correlation of SQM scores with the experimental binding data (Table 2), i.e., Zariquiey structures,^9^ were utilized further. They provide only one modeled conformation (pose) of each ligand in the protein active site. Inspection of these structures revealed multiple issues, such as highly strained geometries of the ligands themselves or clashes between the ligand and the protein. To build better geometries for scoring, we expanded the set of poses by both manual modeling and using docking. The newly generated poses were added to the original structures and thus expanded the pool out of which the final pose for each ligand is selected; see below.

**Table 2:**
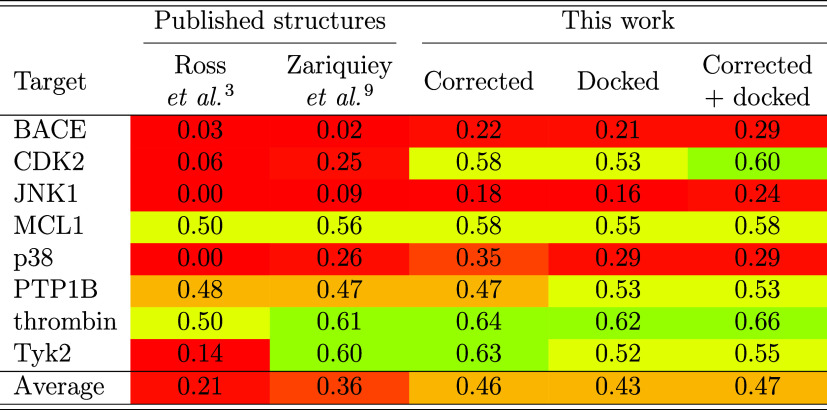
Correlation of SQM2.20’ Score with Experimental Binding Free Energies in the Wang Dataset, Quantified as *R* ^2^, Computed with Previously Published Structures and with the Expanded Sets of Poses Described in This Work

#### 2.2.1 Manual Modifications

Additional plausible conformations for 12 ligands were generated manually, through the rotation of selected torsions to eliminate the steric clashes. Specifically, the ligands designated 13d and 24 of BACE, 20, 28, and 31 of CDK2, 18652 of JNK1, 34, 50, and 52 of MCL1, 2u of p38, 3b of thrombin, and ejm43 of Tyk2 were subjected to this process.

#### 2.2.2 Template-Based Docking

An additional set of poses was generated using template-based docking. For each target, the original protein geometries from Zariquiey structures were used for receptor grid generation, with the center of mass of all the aligned ligands serving as the reference point. The dimensions of the inner box were 12 Å × 12 Å × 12 Å, while those of the outer box were 20 Å × 20 Å × 20 Å. The docking has been carried out using the Glide module^23^ of the Schrödinger 2024–3 suite^24^ in the standard-precision (SP) mode with the OPLS 2005 force field.^25^ The minimum core substructure of all the ligands was defined manually and kept constrained to the coordinates of the ligand from the Zariquiey structure using the MCSSMARTS option with an RMSD tolerance of 0.5 Å. To sample even small conformational changes, further clustering of the poses was disabled. Postdocking minimization was not performed. Up to 100 poses were retained for each ligand. For the BACE protein, crystal structure 4DJX was used instead of 4DJW because it had been shown that it accommodated the studied ligands better (Figure 1).^26^ In case of ligand 2u of p38, the docking generated poses with unfavorable ligand conformations. To alleviate the problem, we generated 73 conformations of the isolated ligand and aligned them to the template without considering the protein.

### 2.3 SQM2.20 Scoring Function

The SQM2.20 scoring function^10^ is a physics-based scoring function based on the latest reparametrization of the PM6-D3H4X method^13–15,17,18^ and the COSMO2 implicit solvent model.^16^ These two methods are crucial for accurate calculation of the leading terms of the binding free energy, namely the gas-phase interaction energy in the P–L complex (Δ*E*_int_) and the change in solvation free energy upon binding (*Δ*Δ*G*_solv_). The remaining terms of the SQM2.20 score account for free energy for the ligand conformational change, protonation state change, and conformational entropy of the ligand, respectively (eq 1). All the terms are used without further empirical scaling, and the methods used to calculate them are not specifically tailored to P–L interactions. For a detailed description of the SQM2.20 scoring function, see ref 10.

1 $$\mathrm{score}=\Delta E_{\mathrm{int}}+\Delta \Delta G_{\mathrm{solv}}+\Delta G_{\mathrm{conf}}\left(L\right)+\Delta G_{H^{+}}-T\Delta S$$

We have observed that in those P–L complexes whose structure was obtained by modeling and the ligand does not fit well into the protein, the deformation energy of the ligand Δ*G*_conf_(L), i.e., the energy difference between the bound and the free ligand conformations, deteriorated the final score. This happened frequently in the Wang data set and thus we use mainly a variant of the score with this term omitted, denoted SQM2.20’. We investigated several strategies for limiting this term to avoid possible artificial errors, but none of these approaches worked significantly better than omitting it altogether, which is a simpler approach free of additional empirical parameters.

The SQM2.20 score was evaluated on SQM/MM optimized P–L complexes. Prior to the SQM/MM optimization of the ligand and its close surroundings, which is an integral part of the scoring protocol,^10^ the P–L complexes in this study were partially optimized at the MM level to resolve steric clashes present in many of the structures. In the case of CDK2, a part of the protein (all residues within 4 Å of the ligands) was optimized to allow the unfavorably positioned Lys89 to adapt to the ligands that were kept frozen during the optimization. For all the other targets, only the ligand was optimized in this step, with the protein structure held frozen.

The MM setup is the same as in the SQM2.20 scoring function, i.e., AMBER ff19SB force field for the protein^27^ and GAFF2 ^28^ for the ligands, combined with the IGB7 implicit solvent.^29^ The PM6 calculations were carried out using MOPAC2016 (http://openmopac.net),^30^ and the D3H4X corrections were added using Cuby4 (http://cuby4.molecular.cz/).^31^ The Cuby4 interface to MOPAC and AmberTools 22 (https://ambermd.org)^22^ facilitated the SQM/MM setup as well as the evaluation of the COSMO2 solvation free energy.^16^ High computational efficiency is achieved by using the MOZYME linear scaling algorithm^21^ for all PM6 calculations; conventional SQM methods are an order of magnitude slower. The mean run time for SQM2.20 scoring (optimization + single point calculations) using surroundings of the protein extending 10 Å around all the aligned ligands (the default model) spanned from 17 to 41 min on a single CPU core (Intel Xeon Gold 6140 2.30 GHz processor) per P–L complex of the Wang data set.

In cases where multiple poses of a ligand had been available, the single representative structure for the scoring was selected as the pose that exhibited the most favorable PM6-D3H4X/COSMO2 total energy of the optimized P–L complex. This selection is strictly physics-based, and excludes any arbitrary factors.

### 2.4 Standard and Machine-Learning Scoring Functions

Fourteen standard SFs and six structure-based machine-learning (ML) SFs were utilized using protocols and best practices from ref 10. Particularly, we used PLP and ChemPLP scoring functions in PLANTS,^32^ AutoDock4,^33^ Autodock Vina,^34,35^ and Vina Radii Optimized function (Vinardo)^36^ in Autodock Suite v.4.2.6, Chemscore (CHS), Goldscore (GS), ChemPLP and ASP^37^ in GOLD v.2022.1.0, X-SCORE’s HPScore, HMS, HSS, and their averaged X-Score,^38^ and DKoes scoring of the Smina fork of AutoDock Vina.^39^ ML methods included standalone algorithms RF-score-VS^40^ Pafnucy^41^ and PIGNet2 ^42^ and standard scoring functions extended with additional ML correction, i.e., Δ_vina_RF_20_,^43^ NNScore2.0,^44^ and Δ_linF9_XGB.^45^ The scoring was performed on the geometries optimized with the SQM2.20 scoring protocol described above. In addition to using optimized SQM/MM geometries (Supporting Table S5), we also evaluated standard and ML-based SFs on ligand poses locally optimized at MM level in rigid protein, as commonly used in SF benchmarking.^46^ This procedure yielded very similar results (see Supporting Table S6) and will not be discussed further. Individual binding sites of the targets were defined as boxes centered on the center of mass of all aligned ligands of the respective P–L series. The results of one more ML-based SF, *K*_DEEP_, were collected from the literature.^47^

### 2.5 MD-Based Binding Free Energies From Literature

Several commercial and open-source MD-based free-energy protocols have been applied to the entire Wang data set by various authors, each employing different methodologies and refinements. The original Schrodinger’s FEP+ alchemical perturbation protocol utilized the OPLS2.1 force field,^3^ while an improved version incorporated the OPLS4 force field and newly prepared ligand and protein input structures (Ross structures).^4^ An alternative automated FEP protocol, implemented in Cresset’s structure-based drug design suite Flare (referred to as Flare FEP), employed the AMBER ff14SB and GAFF2.1 force fields.^5^ Additionally, a FEP protocol combining the AMBER ff14SB force field with alchemical enhanced sampling (ACES), referred to as FEP/ACES, was compared to an earlier approach (FEP/noACES).^6^

A different route to calculating free energies is via thermodynamics integration (TI). It was applied with GAFF1.8 force field without enhanced sampling (AMBER TI)^7^ as well as in GROMACS via nonequilibrium approach using the pmx engine with GAFF2.1 and CGenFF force fields.^8^

As an alternative to the alchemical transformations of ligands in FEP and TI, the alchemical transfer method (ATM),^48^ implemented in the open-source OpenMM MD engine, swaps a ligand in the protein binding site with another ligand in solution. ATM was applied to the entire Wang data set using the GAFF2 force field.^9^

Using different sampling not based on MD, VeraChem’s Mining Minima (VM2) employs a force field combined with an implicit solvent model to calculate binding free energies by evaluating the most stable local energy minima of the protein, ligand, and their complexes.^49^ On the Wang data set, VM2 used AMBER ff14SB and GAFF2.1 force fields with Generalized Born model for optimization and Poisson–Boltzmann Surface Area model in single-point energy calculations.^49^

For the sake of brevity, we do not mention details of other computational studies performed on the Wang data set which either used only a subset of the data set^50,51^ or did not report the absolute binding free energies (Δ*G*_bind_) needed for comparative analysis but only relative ones (ΔΔ*G*_bind_).^52,53^

### 2.6 Analysis of the Results

The SQM2.20 scoring function focuses exclusively on the primary contributors to binding free energy, resulting in a score with a distinct scale. Relative binding free energy methods provide the free energy differences between ligand pairs, ΔΔ*G*_bind_, which can be converted into absolute Δ*G*_bind_ values.^54^ In our study, we employ the squared Pearson correlation coefficient, *R* ^2^, as the primary measure quantifying the correlation between the calculated Δ*G*_bind_ values and the experimentally measured Δ*G*_bind_ values. In the case of anticorrelation (with a negative value of *R*), *R* ^2^ in our tables was set to zero, which enabled a meaningful evaluation of averages across different targets and methods.

## 3 Results and Discussion

### 3.1 SQM2.20 Scoring on the Published Structures

First, the SQM2.20 scoring function was applied to both Zariquiey^9^ and Ross^4^ structures associated with the Wang data set. Although Zariquiey structures for the crystal ligands exhibited similar or higher (except p38 ligand) root-mean-square deviations (RMSD) with respect to the crystal structures (see Table S2 in the SI), the correlation with the experimental binding affinities was found to be superior to that for the Ross structures.^4^ The average RMSD was 0.47 and 0.37 Å, while the average squared Pearson’s coefficient (*R* ^2^) was 0.25 and 0.16 (see Tables S2 and S3 in the Supporting Information), respectively. The correlation improved to 0.36 or 0.21, respectively, when the Δ*G*_conf_(L) term was omitted in the SQM2.20’ variant of the score (see Table 2). This implies that there were inaccuracies in the initial geometries. Indeed, steric clashes between the ligands and proteins, as well as within the ligands themselves, occurred frequently in the input structures. The artifacts present in some of the structures could not be resolved even with geometry optimization, an inherent part of our scoring protocol, and were further adjusted in the specific systems as described in Section 2.2.

### 3.2 SQM2.20 Scoring on the Expanded Conformational Set

In the next step, we addressed the problematic poses by modeling additional conformations, as described in Section 2.2. First, the selected problematic cases were corrected manually. Twelve ligands (at least one in each series except for PTP1B) were selected based on inspection of the geometries and identification of outliers in the SQM2.20 score. An illustrative example of such an intervention is shown in Figure 2. In all cases, the updated structure was accepted only if the resulting SQM energy of the optimized P–L complex was more favorable. For the BACE protein, we also replaced the original protein structure (PDB ID 4DJW) with 4DJX^26^ to achieve more favorable interactions with Tyr132 residue in the flap loop’s closed conformation, see Figure 1 for structure comparison. Interestingly, it was the BACE target for which the protein flexibility, i.e., the crystal structure substitution, had the largest impact on SQM2.20 scoring among all the targets in the PL-REX (see Supporting Table S5 in ref 10). With these modifications, the average *R* ^2^ of SQM2.20’ improved to 0.46. The results for individual targets are listed in Table 2 under the label “Corrected”.

**Figure 1:**
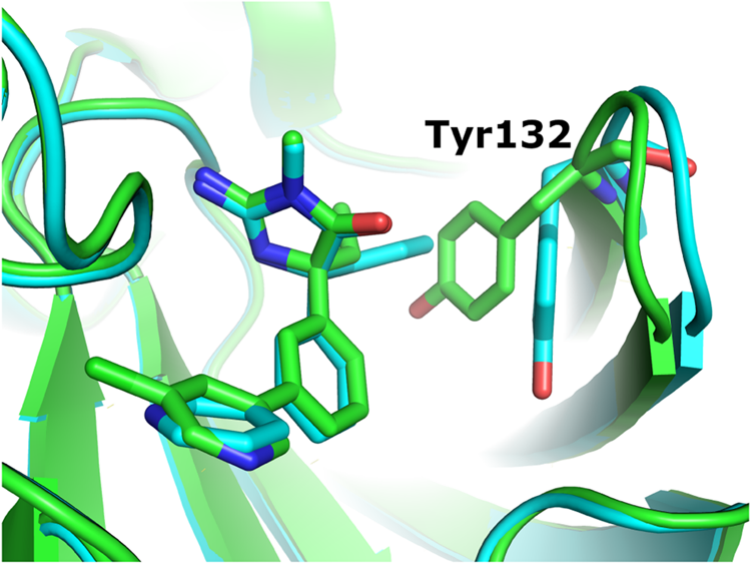
Comparison of the 4DJX and 4DJW structures of BACE1 cocrystals. Carbon atoms are colored green and cyan, respectively.

**Figure 2:**
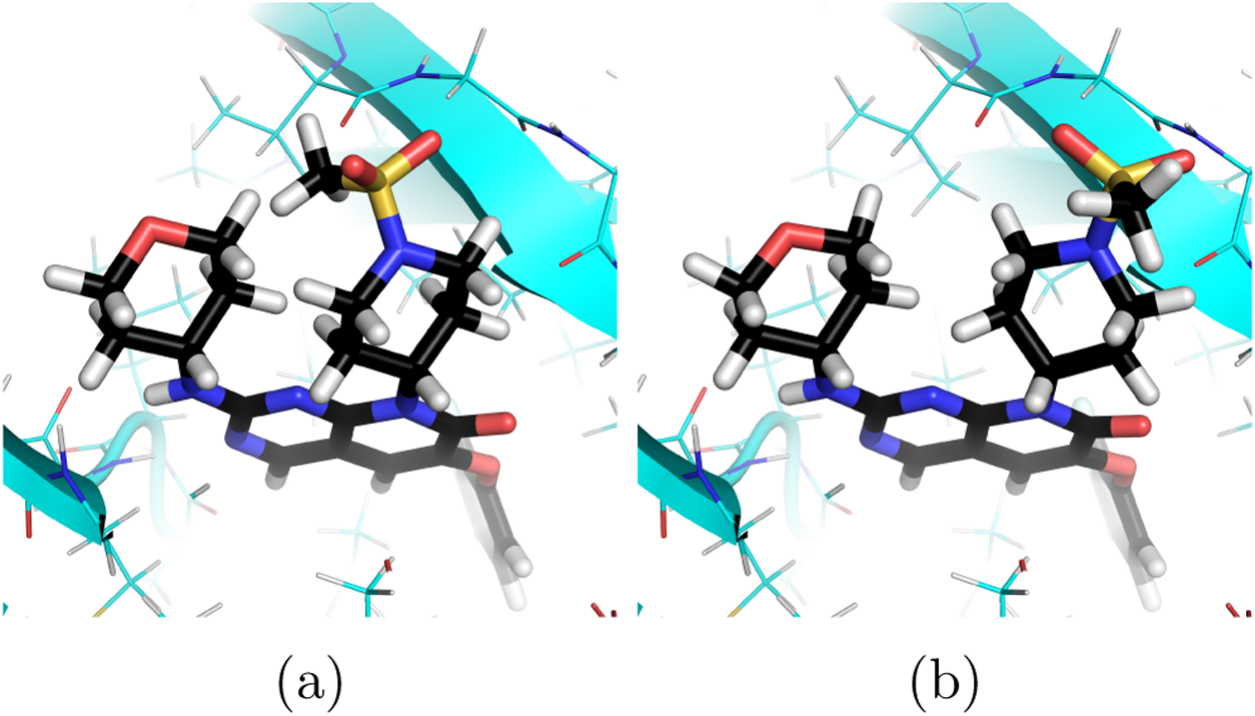
Comparison of (a) the original Zariquiey and (b) manually corrected poses of the 2u ligand of the p38 target. The 1-(methylsulfonyl)­piperidinyl moiety was rotated to avoid intramolecular clashes with the tetrahydropyranyl ring and to form an H-bond between the oxygen atom of the sulfonyl group and the backbone of Ser32. This change improved the total PM6-D3H4X/COSMO2 energy of the optimized geometry by 9.1 kcal/mol.

This improvement, achieved merely by rectifying a few evident errors in the original structures, underscores the critical importance of high-quality input for any single-structure scoring method. Even though the SQM2.20 scoring procedure incorporates geometry optimization, it may still be sensitive to minor details in the input geometry, some of which cannot be rectified by optimizing the structure to the nearest minimum. Conventional scoring functions tend to be less sensitive, perhaps because they do not explicitly account for all hydrogen atoms or because they are parametrized to tolerate certain close contacts. Conversely, MD-based methods equilibrate the structure and sample multiple local minima during the simulation, thereby reducing the impact of the initial structure. Nevertheless, it has been demonstrated that the initial structure can still influence the outcomes of these simulations.^55^ This is also evident when comparing the results of the FEP+ calculations on two sets of structures in the Wang data set.^4^

Second, we automatically generated poses for all the ligands using template-based docking. During this process, each ligand was docked with restraints that kept the common core of the Zariquiey poses fixed. The resulting poses were refined at the SQM level, and the single lowest-energy pose was selected for scoring. This protocol yielded an average *R* ^2^ of 0.43, closely matching the results obtained with manually corrected geometries but without requiring manual intervention.The results for individual targets are reported in Table 2.

The larger pool of poses clearly contains better structures, as they are both lower in energy and improve the scores toward the experimental affinities. However, they did not improve the behavior of the Δ*G*_conf_(L) term, despite the fact that more ligand conformations are sampled. Even in the extended set, the SQM2.20’ SF, which neglects this term, yields better results than the full SQM2.20 score (they can be compared in Tables 2 and S3). This suggests that the problem is probably not caused by the use of erroneous conformations, but rather by an artificial strain on the ligands induced by the protein. This hypothesis is supported by the following analysis: The modeled ligands have a normalized ligand deformation energy (Δ*G*_conf_(L) divided by the number of atoms in the ligand) on average 43% higher than the ligands with experimentally known structure (the values are 0.1057 and 0.0751 kcal/mol/atom, respectively). In contrast to the PL-REX data set, there are no other structures to choose from that would better fit all the ligands. In the PL-REX set, where the most optimal protein structure is selected from the entire series specifically to minimize this strain, the term Δ*G*_conf_(L) generally contributes positively to the score (see Table S4).

However, further analysis of the poses revealed that, in a few cases, the docking failed to recover the optimal binding mode previously found through manual modeling. These failures were primarily due to clashes between the ligand and the protein, which was kept rigid in the docking. To address these problematic cases, we combined all the poses generated from both modeling and docking into a single pool and selected the best pose on the basis of the total SQM energy. With this refined set of structures, the average correlation between SQM2.20’ score and the experimental affinities improved slightly to *R* ^2^ of 0.47. The individual correlations are listed in Table 2 under the label “Corrected + docked”.

Once the obvious issues in the input structures were resolved, the SQM2.20’ scoring was successful, i.e., achieved favorable correlation with *R* ^2^ higher than 0.5 in five targets: CDK2, MCL1, PTP1B, thrombin, and Tyk2. However, in the remaining three targets – BACE, JNK1, and p38 – the correlation was lower, with *R* ^2^ values of 0.29, 0.24 and 0.29, respectively (Table 2). The particularly poor correlation in JNK1 may possibly be attributed to the low resolution of the crystal structure, which has RMSD as high as 3.5 Å (see Table 1). Such a system is unlikely to serve as a reliable benchmark for any method sensitive to the geometry of the complex.

### 3.3 Published MD-Based Free Energy Results

The results of MD-based free energy calculation methods that have been applied to the entire Wang data set and reported Δ*G*_bind_ values, are compared with our results obtained using the SQM2.20’ scoring function. A detailed comparison is presented in Table 3.

**Table 3:**
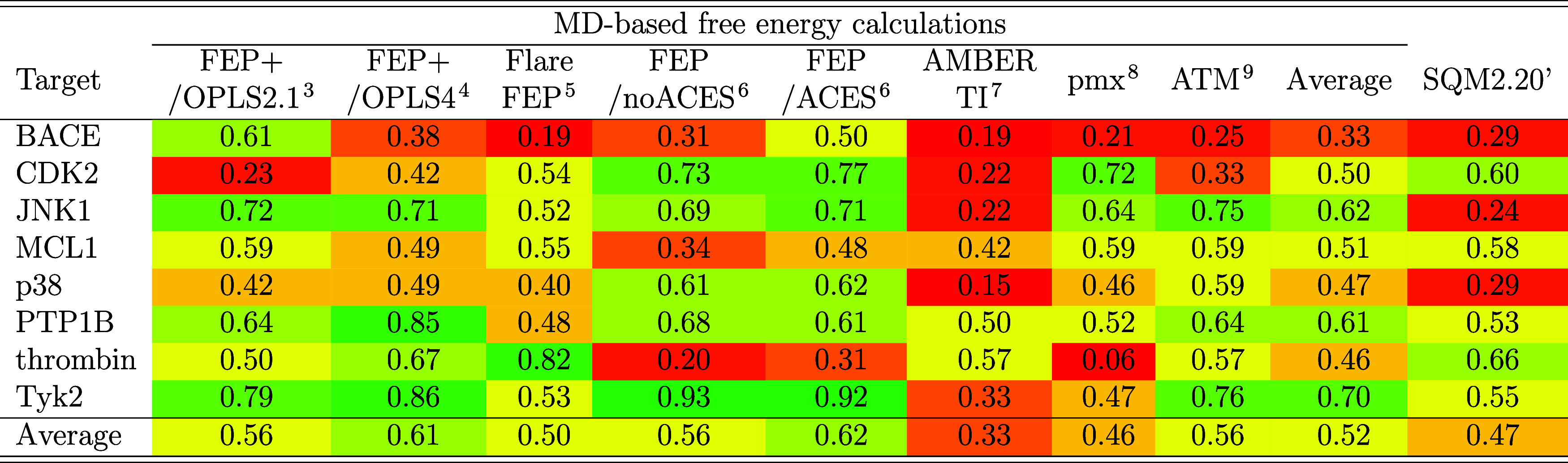
Performance of Various Computational Approaches on the Wang Dataset Expressed as Squared Pearson Correlation Coefficient (*R* ^2^) between the Computed and the Experimental Binding Free Energies^a^

a The last two columns list the average *R* ^2^ of the MD-based methods, and *R* ^2^ of the SQM2.20’ scores. The initial geometries for MD-based approaches were obtained from ref 3, except the FEP+/OPLS4 where they were obtained from ref 4.

In the original study introducing the Wang data set, the FEP+ free energy perturbation protocol was developed and applied using an earlier version of the OPLS force field (v2.1), yielding an average correlation *R* ^2^ of 0.56.^3^ FEP+ demonstrated favorable correlation (*R* ^2^ > 0.5) in six targets, yet exhibited limitations in two, specifically CDK2 and p38, with *R* ^2^ values of 0.23 and 0.42, respectively. An updated methodology incorporating a more recent force field (OPLS4) and new Ross structures led to slightly better overall results (average *R* ^2^ of 0.61).^4^ While the performance on the previously problematic targets improved, the results for others, such as BACE and MCL1, deteriorated (Table 3). Similar results (average *R* ^2^ of 0.64) were obtained by other authors using FEP+ with the OPLSv3 force field and combining free energy estimates from three independent FEP+ runs.^8^ Comparable outcomes were also reported for the Flare FEP method, which achieved an average *R* ^2^ of 0.50. Conventional FEP calculations using the AMBER ff14SB force field (FEP/no ACES) yielded an average *R* ^2^ of 0.56, while the inclusion of enhanced sampling in the alchemical dimension (FEP/ACES) improved the overall results to *R* ^2^ of 0.62, with thrombin being the only challenging target (*R* ^2^ of 0.31).^6^ An early proof-of-concept study on equilibrium TI calculations in AMBER reported an average *R* ^2^ of 0.33 for the Wang data set, with six troublesome targets. The open-source TI implementation, GROMACS nonequilibrium TI (pmx), achieved an average *R* ^2^ of 0.46, with only two troublesome targets (BACE and thrombin). These results were obtained using the GAFF force field, which outperformed the CHARMM generalized force field CGenFF.^8^ (The correlation values reported here are based on refs 5,49 as the original publication^8^ did not provide the Δ*G*_bind_ data.) Another open-source alchemical calculation approach, the Alchemical Transfer Method (ATM), showed similar performance on the Wang data set, with an average *R* ^2^ of 0.56, with two troublesome targets, BACE and CDK2, with an average *R* ^2^ values of 0.25 and 0.33, respectively.^9^

Among the evaluated targets, BACE posed the greatest challenge for MD-based methods, with an average *R* ^2^ of just 0.33. Notably, only the original FEP+ method^3^ and the FEP/ACES protocol,^6^ which incorporated enhanced conformational sampling, reached *R* ^2^ values above 0.5. The SQM2.20’ results (*R* ^2^ of 0.29) were comparable to those of most MD-based methods. In contrast, the JNK1 target, where SQM2.20’ performed the worst (*R* ^2^ of 0.24), was well-described by the majority of MD-based methods, with the exception of AMBER TI. This highlights the ability of MD-based methods to handle systems even when relying on medium-resolution crystal structures. On the other hand, the variability in performance of MD-based methods across individual targets underscores the fact that the accuracy of the free-energy calculations depends on more than just the simulation protocol. Factors such as force field parameters, sampling time, and simulation conditions collectively influence the reliability and precision of the calculated binding free energies.

Regarding the computational costs, MD-based methods generally require significantly higher computational resources compared to the SQM2.20 method, even if we account for the evaluation of multiple poses of each ligand at the SQM level. While SQM2.20 operates efficiently with a runtime of only about 30 min per P–L complex on a single CPU core (this includes both the optimization of the complex and the calculation of the score), MD-based methods like FEP+ and AMBER-TI involve much longer simulation times, often spanning several hours per run and leveraging high-performance but costly GPUs. The computational cost of different MD-based free-energy approaches is difficult to compare directly, as they differ in many factors, such as sampling strategy, algorithmic details, convergence, hardware variability, and scalability. However, the benchmarks reported in the literature might give an overall picture of the computational efficiency of these methods, even though calculated on different systems. For example, the authors of Flare’s FEP implementation reported that calculating the binding free energy for a ligand pair requires a minimum of 1.7 h if parallelized over 18 windows on NVIDIA GTX2070 GPUs. This estimate was made on TYK2 and CHK1 systems, containing 288 and 252 amino acids, requiring on average 80 min for a P–L complex and 18 min for the solvated ligand for a 4 ns 9 λ-window perturbation.^5^ Similar timing was reported for Schrodinger FEP+ Desmond simulations on BACE1 system (401 amino acids), taking around 2 h to sample a single perturbation on 4 NVIDIA Tesla K80 GPUs (i.e., 86 and 34 min on average for a 1 ns 12 λ-window perturbation for complex and solvent, respectively).^56^ In comparison, AMBER-TI with linear alchemical mixing, benchmarked on a ligand pair of the clotting factor Xa (244 amino acids), demonstrated a strong dependence on hardware architecture. Calculations using 4 ns λ-windows required 12 h on an NVIDIA Tesla K80 GPU and 2.5 h on an NVIDIA GTX980 GPU.^57^ The implementation in AMBER18 showed further performance improvements, requiring only 48 min on a single GTX1080 GPU.^58^ Notably, these calculations were at least 2 orders of magnitude slower than the GPU when performed on a single CPU.

### 3.4 Conventional, Machine-Learning, and Other Scoring Methods

For comparison, in addition to the previously published results (i.e., Glide SP,^3^ *K*_DEEP_,^47^ MM-GB/SA,^3^ and VM2 ^49^), we scored the updated Wang data set using a wide range of conventional SFs and structure-based ML approaches (Figure 3 and Table S5). The results demonstrated the limitations of SFs, which, despite delivering results within seconds, often fell short in predictive accuracy, as their averaged *R* ^2^ over the Wang data set ranged from 0.20 to 0.36. The best performing SF was Gold ChemPLP followed by Autodock4, with average *R* ^2^ of 0.36 and 0.30, respectively. Both methods were successful only in two systems, Gold ChemPLP in PTP1B and CDK2 (with *R* ^2^ of 0.64 and 0.49, respectively) and Autodock4 in thrombin and PTP1B (with *R* ^2^ of 0.66 and 0.46, respectively; for more details, see Table S5 in SI). The average performance of seven ML-based or ML-augmented SFs (yellow in Figure 3) was only marginally better than that of the conventional SFs, with more varied results. Δ_vina_RF_20_ (*R* ^2^ = 0.37) and PIGNet2 (*R* ^2^ = 0.43) slightly surpass even the best conventional SF. PIGNet2 performs exceptionally well in this data set. However, it does not appear to be highly transferable, as evidenced by its average *R* ^2^ of 0.34 in the PL-REX set,^10^ which is comparable to the performance of the conventional SFs in that context.

**Figure 3:**
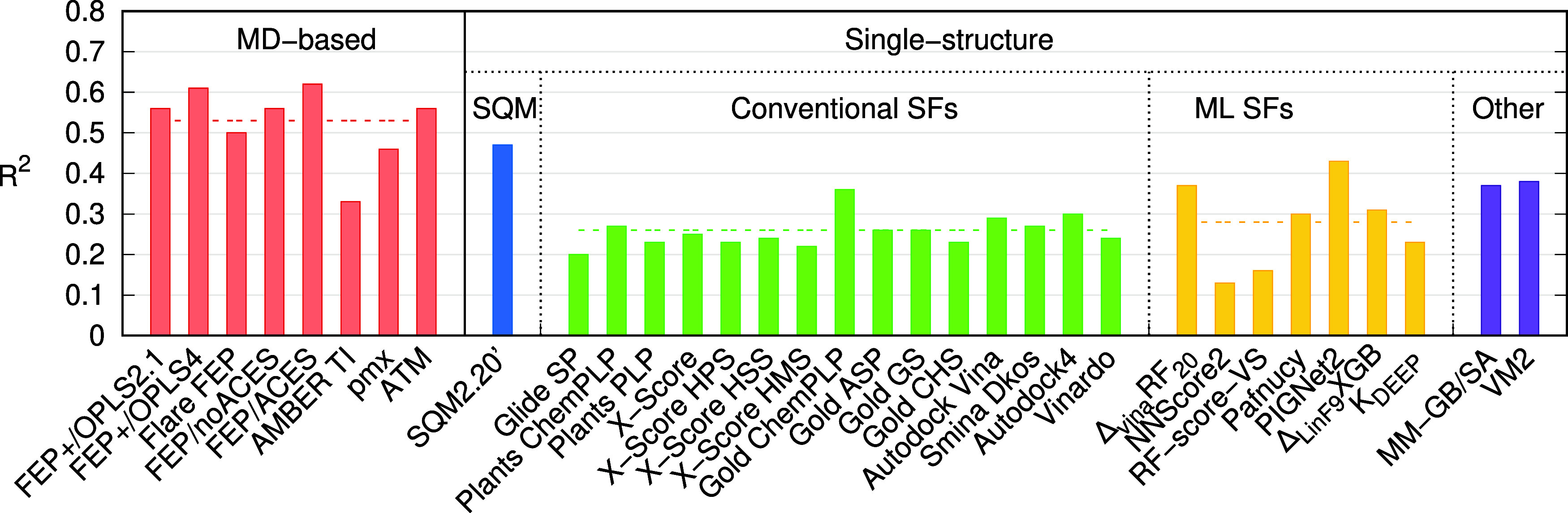
Averaged squared Pearson correlations (*R* ^2^) over the Wang data set. The dashed lines show the average in each class of methods. The results of the MD-based methods, Glide SP, *K*_DEEP_, MM-GB/SA, and VM2, are taken from the literature as described in the text.

On the other hand, the previously published results of two other methods (MM-GB/SA and VM2, purple in Figure 3) have demonstrated better performance. The rigid-receptor MM-GB/SA approach^3^ was successful in the case of three targets, i.e., PTP1B, thrombin, and Tyk2, with *R* ^2^ values of 0.45, 0.86, and 0.62, respectively, leading to an average *R* ^2^ of 0.37. Since the MM-GB/SA covers similar terms as SQM2.20, the difference between the two methods highlights the higher accuracy of SQM compared to MM force field, which was also demonstrated in the PL-REX data set.^10^ Similarly, the VeraChem’s mining minima (VM2) approach,^49^ which explores low-energy conformations/minima using the AMBER ff14SB and GAFF2 force fields, achieved an average *R* ^2^ of 0.38. Particularly, the VM2 with default settings was successful in the case of four targets—BACE, CDK2, JNK1, and thrombin - with *R* ^2^ values of 0.45, 0.77, 0.45, and 0.55, respectively. Additional procedural variations of VM2 reported in the original paper did not lead to significant improvements, with *R* ^2^ values ranging from 0.25 to 0.41.^49^ The VM2 calculations are significantly faster than typical explicit solvent free-energy MD simulations, averaging about 22 min wall-clock time per ligand on 12 cores of an AMD EPYC 7452 2.35 GHz CPU.^49^ Here, the SQM2.20 method, with similar timing to the VM2 method, i.e., on average 27 min per ligand but on a single CPU core, performs better on average (*R* ^2^ of 0.47) despite lacking the conformational sampling.

### 3.5 Single-Structure vs MD-Based Methods Comparison

The results compiled in this work also allow for a more general comparison of the two fundamentally different approaches, the single-structure methods working with one representative geometry of the system, and free-energy calculations based on MD simulations working with ensembles of geometries. To identify general trends, we averaged the results within each class for every target (Figure 4 and Table S7). Single-structure scoring methods are represented by approximate conventional SFs, where we take the average of 15 available SFs (see Table S5), as well as by the SQM2.20’ scoring, which is analyzed separately. We have excluded the ML-based SFs from this analysis since they display patterns akin to the conventional ones, albeit with less pronounced distinctions between the systems. The detailed results are available in the Supporting Information, Table S7. For MD-based methods, we consider both the average correlation (as reported in Table 3) and the best-performing method within this class, FEP/ACES. The results are summarized in Figure 4.

**Figure 4:**
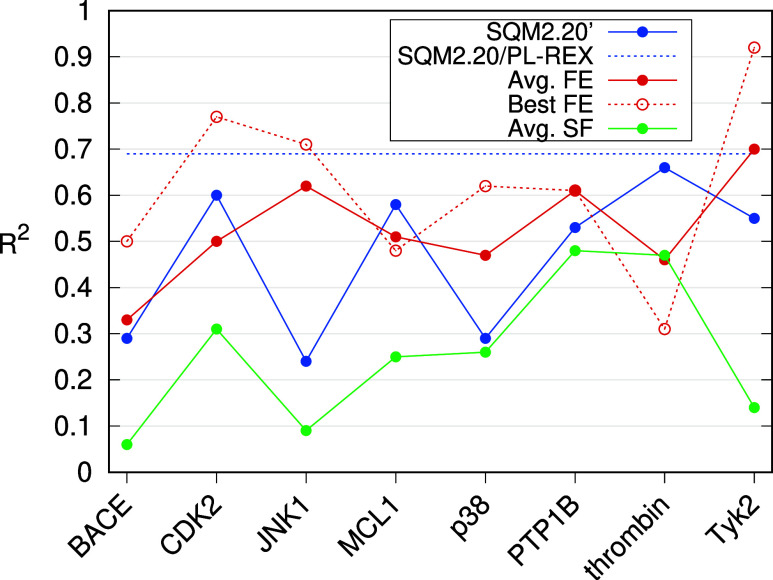
Performance (correlation with the experimental binding affinity) of the studied approaches: SQM scoring (blue), MD-based free energy calculations (red), and conventional scoring functions (green). For SQM scoring, the average performance in the PL-REX data set is shown for comparison. For free energy methods, the average (solid line) and the best method, FEP/ACES, (dotted line) are plotted.

We observe a significant trend across the eight targets: the single-structure methods (both the conventional SFs and SQM2.20’) exhibit similar behavior. Despite differences in absolute accuracy, they tend to fail or succeed in the same systems, as reflected in the high correlation between these results (*R* = 0.67). In contrast, no such relationship is observed between the MD-based free energy methods and SQM2.20’, even though their overall performance is comparable (with *R* ^2^ averaged over the Wang data set being 0.52 and 0.47, respectively). The correlation between their results has *R* of only 0.10. This indicates that some of the failures of the single-structure methods can be explained by their inherent properties. Excluding the BACE target, where neither method performs satisfactorily, the failure of all single-structure methods in JNK1 is particularly notable. A plausible explanation for this is the limited resolution of the crystal structure at 3.5 Å, the lowest in the data set. It is likely that a reliable model for scoring cannot be based on this structure, but MD simulations can refine it. Additionally, the low resolution may be associated with the higher flexibility of the protein, which is not accounted for in single-structure scoring but can be sampled by MD. A similar, albeit less pronounced, issue is observed with the p38 protein. Although the crystal resolution is adequate here (1.8 Å), the ligand itself is poorly resolved, with the lowest RSCC (0.86) in the data set. In addition, the crystallographic structure of near-native protein–ligand complexes may not be available at all, which is often the case in practical applications. Therefore, SQM2.20 scoring is mainly applicable in the hit-to-lead optimization stage.

Our assertion that a dependable structural model is essential for the effective scoring with single-structure methods is further corroborated by the results previously obtained in the PL-REX data set, where the input structures are significantly more reliable (see Table S1 in the Supporting Information). There, the SQM2.20 scoring achieves an average *R* ^2^ of 0.69 and does not fail in any of the ten targets. This performance is on par with the results of the best MD-based method, FEP/ACES, in the Wang data set with an average *R* ^2^ of 0.62 (these data are also plotted in Figure 4).

## 4 Conclusions

In a thorough and systematic manner, we have recalibrated the Wang data set, which consists of eight P–L complex series, utilizing the advanced SQM2.20 scoring function based on semiempirical quantum-mechanical calculations. We compared the results with a wide range of approximate scoring functions. Additionally, we included data from eight different state-of-the-art MD-based free-energy methods and two other computational techniques from the literature.

Our preliminary analysis indicated that certain modeled P–L complex structures within the Wang data set are inadequate for single-structure scoring. To address this limitation, we generated an extensive pool of ligand conformations through a combination of manual modeling and template-based docking. From this comprehensive pool, we selected a new set of representative poses based on their SQM energy values. This expanded repository of poses for the Wang data set accompanies this publication, enhancing its utility. With this refined ensemble of modeled structures, the SQM2.20 scoring function with omitted Δ*G*_conf_, denoted as SQM2.20’, achieved an average *R* ^2^ of 0.47 when correlated with the experimental binding free energies across the eight targets. This result surpasses those of the conventional scoring functions (average *R* ^2^ of 0.26) and approaches the performance of MD-based free energy methods (average *R* ^2^ of 0.52). However, it still falls short of the best MD-based methods which achieve *R* ^2^ values of up to 0.62. Nevertheless, the SQM2.20 scoring function yields these results in a fraction of the time of the MD simulations, averaging 27 min per protein–ligand complex on a single CPU core. This efficiency makes it a viable option for practical applications where computational cost is a key consideration.

Further analysis of the shared trends between single-structure and MD-based approaches reveals that, for certain targets, the efficacy of single-structure methods is limited by the quality of the input structures, such as the low resolution of crystallographic data. In contrast, MD simulations demonstrate greater robustness in this aspect. This observation explains why the SQM2.20 scoring function performed better in the PL-REX data set, which features more reliable initial geometries, compared to the present data set.

In summary, when adequate input structures are available, SQM2.20 scoring has been shown to be competitive with MD-based methods in predicting protein–ligand binding affinities. This is accompanied by a substantial reduction in computation time, thereby establishing SQM2.20 as a powerful tool in computational chemistry, particularly when considering the balance between accuracy and computational efficiency.

## Supplementary Material



## Data Availability

The SQM/MM-optimized structures of the P-L complexes for both the variants of the data set, as well as for all the newly generated and corrected poses are available in the repository https://github.com/Honza-R/Wang_dataset_SQM. The repository also contains all the scores computed on these structures and discussed in the paper.

## Notes

Composition and features of the benchmark protein–ligand data set PL-REX; root-mean-square ligands’ deviations of Zariquiey and Ross structures with respect to the X-ray structures; correlation of SQM2.20 score with experimental binding free energies in the Wang data set; correlation of SQM2.20 and SQM2.20’ scores in the PL-REX data set; correlations calculated on SQM/MM-optimized P-L geometries; correlations calculated on MM-optimized ligands in rigid protein geometry; and the averaged performance of studied approaches within target classes (PDF)
