# Supplementary material for: Comparative Analysis of Quantum-Mechanical and Standard Single-Structure Protein–Ligand Scoring Functions with MD-Based Free Energy Calculations
Source: J Chem Inf Model. 2025 Jul 19;65(15):8127–36. doi: 10.1021/acs.jcim.5c00604 (PMC12344775; doi:10.1021/acs.jcim.5c00604)
Supplement: Supplementary file 1 [file ci5c00604_si_001.pdf]

Supporting Information for:

Comparative Analysis of Quantum-Mechanical and Standard Single-Structure Protein-Ligand Scoring Functions with MD-Based Free Energy Calculations

Mehran Jalaie,[†,¶] Jindřich Fanfrlík,[‡,¶] Adam Pecina,[‡] Martin Lepšík,[‡] and Jan Řezáč[*,‡]

†*Pfizer Worldwide Research and Development, Oncology Medicinal Chemistry, Pfizer, La Jolla, California 92121, United States*

‡*Institute of Organic Chemistry and Biochemistry of The Czech Academy of Sciences, 160 00 Prague, Czech Republic*

¶*Contributed equally to this work*

***e-mail: rezac@uochb.cas.cz**

June 25, 2025

Supplementary Tables

Table S1: Composition and features of the benchmark protein–ligand dataset PL-REX. The ligand similarity is expressed as the average of the Tanimoto coefficients computed for each pair of ligands.

Target	Protein	Ligands/ Crystals	pKi range	Ligand similarity	Crystal used/resolution/ RSCC
01-CA2	Carbonic anhydrase II	10/10	2.2	0.32	5NXG/1.2/0.99
02-HIV-PR	HIV-1 protease	22/12	5.1	0.51	2AQU/2.0/0.94
03-CK2	*Zea mays* casein kinase 2	16/16	1.9	0.32	3KXN/2.0/0.88
04-AR	Aldose reductase	14/14	2.8	0.47	4XZH/1.0/0.98
05-Cath-D	Cathepsin D	10/3	3.5	0.71	6QCB/1.6/0.95
06-BACE1	Beta-secretase 1	16/16	3.6	0.48	5QCZ/2.3/0.92
07-JAK1	Janus kinase 1	12/12	3.4	0.55	4IVD/1.9/0.97
08-Trypsin	*Bovine* trypsin	15/15	4.4	0.45	1K1I/2.2/0.96
09-CDK2	Cyclin-dependent kinase 2	31/31	3.6	0.65	3R9H/2.1/0.92
10-MMP12	Matrix metallopeptidase 12	18/18	3.9	0.47	3EHY/1.9/0.98

Table S2: The root-mean-square deviation in Å of the ligand non-hydrogen atoms of Zariquiey[1] and Ross[2] structures with respect to the X-ray structures

		Structures	
Target	Crystal used	Zariquiey	Ross
BACE	4DJW	1.00	1.00
CDK2	1H1Q	0.26	0.20
JNK1	2GMX	0.61	0.47
MCL1	4HW3	0.63	0.10
p38	3FLY	0.19	0.32
PTP1B	2QBS	0.33	0.32
thrombin	2ZFF	0.55	0.39
Tyk2	4GIH	0.21	0.15

Table S3: Correlation of SQM2.20 score with experimental binding free energies in the Wang dataset, quantified as R^2.

Target	Published structures		This work		
	Ross *et al.*[3]	Zariquiey *et al.*[1]	Corrected	Docked	Corrected + docked
BACE	0.05	0.07	0.13	0.27	0.33
CDK2	0.00	0.14	0.33	0.32	0.41
JNK1	0.00	0.01	0.08	0.01	0.04
MLC1	0.51	0.48	0.53	0.44	0.50
p38	0.00	0.22	0.31	0.26	0.26
PTP1B	0.21	0.17	0.17	0.32	0.32
thrombin	0.37	0.38	0.39	0.42	0.43
Tyk2	0.17	0.55	0.65	0.54	0.62
Average	0.16	0.25	0.32	0.32	0.36

Table S4: Correlation of SQM2.20 and SQM2.20' scores with the experimental binding free energies in the PL-REX dataset, quantified as R^2.

Target	SQM2.20	SQM2.20'
01-CA2	0.67	0.65
02-HIV-PR	0.75	0.78
03-CK2	0.81	0.76
04-AR	0.70	0.68
05-Cath-D	0.66	0.58
06-BACE1	0.63	0.39
07-JAK1	0.56	0.63
08-Trypsin	0.75	0.84
09-CDK2	0.61	0.55
10-MMP12	0.74	0.81
Average	0.69	0.67

Table S5: Correlation (squared Pearson coefficient, R^2) of the scores with experimental binding free energies, calculated on SQM/MM-optimized P-L geometries. The results of GlideSP and MM-GB/PBSA were taken from Ref.[3], K_{DEEP} from Ref.[4] and VM2 method from Ref.[5]; note that these values were obtained using different geometries.

Target	PLANTS ChemPLP	PLANTS PLP	X-Score	X-Score HPS	X-Score HSS	X-Score HMS	GOLD ChemPLP	GOLD ASP
BACE	0.04	0.07	0.00	0.02	0.02	0.00	0.32	0.00
CDK2	0.22	0.12	0.26	0.40	0.02	0.27	0.49	0.53
JNK1	0.10	0.18	0.00	0.00	0.00	0.00	0.28	0.08
MCL1	0.29	0.24	0.27	0.17	0.46	0.23	0.18	0.07
p38	0.37	0.40	0.28	0.27	0.35	0.20	0.32	0.24
PTP1B	0.22	0.35	0.53	0.53	0.55	0.48	0.64	0.55
thrombin	0.62	0.44	0.64	0.46	0.55	0.61	0.37	0.49
Tyk2	0.25	0.02	0.00	0.01	0.00	0.00	0.28	0.08
Average	0.27	0.23	0.25	0.23	0.24	0.22	0.36	0.26

Target	GOLD GS	GOLD CHS	Autodock Vina	Smina Dkos	Autodock4	Vinardo	Glide SP	$\Delta_{Vina}RF_{20}$
BACE	0.00	0.04	0.20	0.03	0.00	0.20	0.00	0.24
CDK2	0.30	0.42	0.40	0.51	0.40	0.29	0.00	0.36
JNK1	0.01	0.31	0.12	0.00	0.18	0.00	0.06	0.49
MCL1	0.40	0.28	0.22	0.18	0.29	0.15	0.35	0.30
p38	0.17	0.03	0.37	0.34	0.31	0.23	0.02	0.46
PTP1B	0.46	0.38	0.56	0.63	0.46	0.50	0.30	0.63
thrombin	0.48	0.31	0.40	0.46	0.66	0.27	0.28	0.19
Tyk2	0.29	0.09	0.06	0.02	0.12	0.23	0.62	0.28
Average	0.26	0.23	0.29	0.27	0.30	0.24	0.20	0.37

Target	NNScore2	RF-score-VS	Pafnucy	PIGNet2	Δ_{LinF9}XGB	K_{DEEP}	MM-GB/SA	VM2
BACE	0.02	0.00	0.04	0.32	0.26	0.00	0.00	0.45
CDK2	0.13	0.03	0.01	0.58	0.01	0.48	0.00	0.77
JNK1	0.04	0.29	0.24	0.01	0.00	0.48	0.42	0.45
MCL1	0.25	0.06	0.41	0.60	0.37	0.12	0.18	0.32
p38	0.03	0.29	0.42	0.33	0.53	0.13	0.44	0.14
PTP1B	0.10	0.03	0.62	0.49	0.37	0.34	0.45	0.00
thrombin	0.00	0.57	0.67	0.65	0.58	0.34	0.86	0.55
Tyk2	0.47	0.01	0.01	0.44	0.37	0.00	0.62	0.36
Average	0.13	0.16	0.30	0.43	0.31	0.23	0.37	0.38

Table S6: Correlation (squared Pearson coefficient, R^2) of the scores with experimental binding free energies, calculated on MM-optimized ligands in rigid protein geometry. The results of GlideSP and MM-GB/PBSA were taken from Ref.[3], K_{DEEP} from Ref.[4] and VM2 method from Ref.[5]; note that these values were obtained using different geometries.

Target	PLANTS ChemPLP	PLANTS PLP	X-Score	X-Score HPS	X-Score HSS	X-Score HMS	GOLD ChemPLP	GOLD ASP
BACE	0.11	0.19	0.02	0.00	0.02	0.11	0.06	0.00
CDK2	0.17	0.16	0.11	0.01	0.25	0.06	0.27	0.31
JNK1	0.04	0.06	0.51	0.23	0.48	0.47	0.06	0.24
MCL1	0.20	0.25	0.25	0.13	0.43	0.23	0.17	0.04
p38	0.33	0.36	0.27	0.24	0.33	0.19	0.33	0.33
PTP1B	0.12	0.39	0.62	0.57	0.58	0.62	0.56	0.54
thrombin	0.54	0.31	0.62	0.44	0.52	0.64	0.44	0.43
Tyk2	0.55	0.58	0.00	0.07	0.01	0.13	0.69	0.58
Average	0.26	0.29	0.30	0.21	0.32	0.31	0.32	0.31

Target	GOLD GS	GOLD CHS	Autodock Vina	Smina Dkos	Autodock4	Vinardo	Glide SP	$\Delta_{Vina}RF_{20}$
BACE	0.06	0.01	0.22	0.01	0.00	0.28	0.25	0.23
CDK2	0.56	0.25	0.28	0.24	0.12	0.24	0.04	0.26
JNK1	0.08	0.44	0.25	0.09	0.04	0.53	0.34	0.09
MCL1	0.41	0.12	0.20	0.19	0.34	0.17	0.05	0.22
p38	0.43	0.05	0.32	0.35	0.32	0.08	0.34	0.46
PTP1B	0.46	0.45	0.49	0.60	0.17	0.29	0.28	0.48
thrombin	0.60	0.60	0.46	0.40	0.60	0.36	0.49	0.30
Tyk2	0.59	0.24	0.34	0.11	0.28	0.68	0.55	0.53
Average	0.40	0.27	0.32	0.25	0.24	0.33	0.29	0.32

Target	NNScore2	RF-score-VS	Pafnucy	PIGNet2	Δ_{LinF9}XGB	K_{DEEP}	MM-GB/SA	VM2
BACE	0.02	0.00	0.14	0.23	0.25	0.00	0.00	0.45
CDK2	0.06	0.04	0.20	0.22	0.33	0.48	0.00	0.77
JNK1	0.18	0.29	0.60	0.01	0.39	0.48	0.42	0.45
MCL1	0.13	0.09	0.20	0.59	0.36	0.12	0.18	0.32
p38	0.12	0.43	0.27	0.32	0.52	0.13	0.44	0.14
PTP1B	0.12	0.00	0.51	0.40	0.37	0.34	0.45	0.00
thrombin	0.32	0.52	0.72	0.63	0.58	0.34	0.86	0.55
Tyk2	0.22	0.10	0.07	0.53	0.50	0.00	0.62	0.36
Average	0.15	0.18	0.34	0.37	0.41	0.23	0.37	0.38

Table S7: The averaged performance of studied approaches (squared Pearson coefficient, R^2) within target classes. "Avg. FE" stands for an average of all MD-based free energy calculations, "Best FE" stands for FEP/ACES method, "Avg. SF" and Avg. ML-SF stands for the average performance of all conventional and ML-based scoring functions, respectively.

Target	SQM2.20'	Avg. FE	Best FE	Avg. SF	Avg. ML-SF
BACE	0.29	0.33	0.5	0.06	0.12
CDK2	0.6	0.5	0.77	0.31	0.23
JNK1	0.24	0.62	0.71	0.09	0.29
MCL1	0.58	0.51	0.48	0.25	0.24
p38	0.29	0.47	0.62	0.26	0.32
PTP1B	0.53	0.61	0.61	0.48	0.32
thrombin	0.66	0.46	0.31	0.47	0.49
Tyk2	0.55	0.7	0.92	0.14	0.28
Average	0.47	0.52	0.62	0.26	0.29

References

(1) Zariquiey, F. S.; Perez, A.; Majewski, M.; Gallicchio, E.; De Fabritiis, G. Validation of the Alchemical Transfer Method for the Estimation of Relative Binding Affinities of Molecular Series. *J. Chem. Inf. Model.* **2023**, *63*, 2438–2444.

(2) Ross, G. A.; Lu, C.; Scarabelli, G.; Albanese, S. K.; Houang, E.; Abel, R.; Harder, E. D.; Wang, L. The maximal and current accuracy of rigorous protein-ligand binding free energy calculations. *Commun. Chem.* **2023**, *6*, 222.

(3) Wang, L.; Wu, Y.; Deng, Y.; Kim, B.; Pierce, L.; Krilov, G.; Lupyan, D.; Robinson, S.; Dahlgren, M. K.; Greenwood, J.; Romero, D. L.; Masse, C.; Knight, J. L.; Steinbrecher, T.; Beuming, T.; Damm, W.; Harder, E.; Sherman, W.; Brewer, M.; Wester, R.; Murcko, M.; Frye, L.; Farid, R.; Lin, T.; Mobley, D. L.; Jorgensen, W. L.; Berne, B. J.; Friesner, R. A.; Abel, R. Accurate and Reliable Prediction of Relative Ligand Binding Potency in Prospective Drug Discovery by Way of a Modern Free-Energy Calculation Protocol and Force Field. *J. Am. Chem Soc.* **2015**, *137*, 2695–2703.

(4) Jiménez, J.; Škalič, M.; Martínez-Rosell, G.; De Fabritiis, G. KDEEP: Protein–Ligand Absolute Binding Affinity Prediction via 3D-Convolutional Neural Networks. *Journal of Chemical Information and Modeling* **2018**, *58*, 287–296.

(5) Gilson, M. K.; Stewart, L. E.; Potter, M. J.; Webb, S. P. Rapid, Accurate, Ranking of Protein–Ligand Binding Affinities with VM2, the Second-Generation Mining Minima Method. *J. Chem. Theory Comput.* **2024**, *20*, 6328–6340.
